# Changes of Proteases, Antiproteases, and Pathogens in Cystic Fibrosis Patients' Upper and Lower Airways after IV-Antibiotic Therapy

**DOI:** 10.1155/2015/626530

**Published:** 2015-06-21

**Authors:** Ulrike Müller, Julia Hentschel, Wibke K. Janhsen, Kerstin Hünniger, Uta-Christina Hipler, Jürgen Sonnemann, Wolfgang Pfister, Klas Böer, Thomas Lehmann, Jochen G. Mainz

**Affiliations:** ^1^Department of Pediatrics, Cystic Fibrosis Center, Jena University Hospital, 07740 Jena, Germany; ^2^Septomics Research Center, Friedrich Schiller University, 07745 Jena, Germany; ^3^Leibniz Institute for Natural Product Research and Infection Biology, Hans Knoell Institute, Jena, Germany; ^4^Department of Dermatology, Jena University Hospital, 07740 Jena, Germany; ^5^Department of Pediatric Hematology and Oncology, Jena University Hospital, 07740 Jena, Germany; ^6^Institute of Medical Microbiology, University of Jena, 07740 Jena, Germany; ^7^Institute for Clinical Chemistry and Laboratory Diagnostics, Jena University Hospital, 07740 Jena, Germany; ^8^Institute of Medical Statistics, Computer Sciences and Documentation, Jena University Hospital, 07740 Jena, Germany

## Abstract

*Background*. In cystic fibrosis (CF) the upper (UAW) and lower airways (LAW) are reservoirs for pathogens like *Pseudomonas aeruginosa*. The consecutive hosts' release of proteolytic enzymes contributes to inflammation and progressive pulmonary destruction. Objectives were to assess dynamics of protease : antiprotease ratios and pathogens in CF-UAW and LAW sampled by nasal lavage (NL) and sputum before and after intravenous- (IV-) antibiotic therapy. *Methods*. From 19 IV-antibiotic courses of 17 CF patients NL (10 mL/nostril) and sputum were collected before and after treatment. Microbiological colonization and concentrations of NE/SLPI/CTSS (ELISA) and MMP-9/TIMP-1 (multiplex bead array) were determined. Additionally, changes of sinonasal symptoms were assessed (SNOT-20). *Results*. IV-antibiotic treatment had more pronounced effects on inflammatory markers in LAW, whereas trends to decrease were also found in UAW. Ratios of MMP-9/TIMP-1 were higher in sputum, and ratios of NE/SLPI were higher in NL. Remarkably, NE/SLPI ratio was 10-fold higher in NL compared to healthy controls. SNOT-20 scores decreased significantly during therapy (*P* = 0.001). *Conclusion*. For the first time, changes in microbiological patterns in UAW and LAW after IV-antibiotic treatments were assessed, together with changes of protease/antiprotease imbalances. Delayed responses of proteases and antiproteases to IV-antibiotic therapy were found in UAW compared to LAW.

## 1. Introduction

Cystic fibrosis (CF) is the most common lethal autosomal recessive inherited chronic disease in the Caucasian population and is caused by mutations in the cystic fibrosis transmembrane conductance regulator (*CFTR, *7q31). Defective ion channels lead to production of viscous secretions from exocrine glands. In CF, the innate immunity is ineffective because of impaired mucociliary clearance and immune cellular causes [1]. This allows chronic pathogen colonization and in airway, inflammation which results in progressive pulmonary destruction as main reason for increased morbidity and mortality in CF [2–5]. Pathogen colonization with* Staphylococcus (S.) aureus *and* Haemophilus influenzae *commonly begins in the first few months of life [6]. Later on, gram-negative organisms dominate, as* Pseudomonas (P.) aeruginosa* which chronically colonizes the lungs of 70–80% of adult CF patients [7].* P. aeruginosa* enhances inflammation in CF airways, for example, by causing the release of different proinflammatory and immunological active components, promoting secretion of mucus and impairing ciliary function [8].

Inflammation in CF airways is neutrophil-dominated; thus high levels of the proteolytic enzyme neutrophil elastase (NE) and oxidants can be found in the airway surface liquid [13]. At the same time enzymes with protective function in CF airways like *α*_1_-antitrypsin and secretory leukocyte protease inhibitor (SLPI) can be inactivated by NE [39]. Furthermore, NE can enhance pulmonary inflammation and destruction by degrading extracellular matrix components. NE also serves as a biomarker for inflammation in CF [2, 19, 40–42]. The release of NE by neutrophils can be stimulated upon different cytokines and chemoattractants, for example, TNF and IL-8 [43]. High concentrations of NE and IL-8 in the airway surface liquid overwhelm and inactivate the antiprotease defense system, deranging the balance of proteases and antiproteases which is required for equilibration of defense mechanisms and prevention of tissue damage [8]. Recently, concentrations of NE in CF patients' lower were found to be elevated, compared to concentrations in the upper airways (LAW/UAW) [16]. In the UAW especially the serine protease SLPI is a major antagonist of NE. Contained in mucosal lining fluids SLPI is produced by macrophages, neutrophils, and epithelial cells of the respiratory and alimentary tract. Due to its high cationicity, SLPI can disrupt microbial membranes, affecting opportunistic pathogens in the lungs such as* S. aureus *and* P. aeruginosa* as well as skin pathogens, for example,* S. epidermidis *and* Candida albicans*, to become established [12, 44]. Increased concentrations of SLPI can be found in infection, for example, in pneumonia, whereas downregulation is triggered by interferon-gamma (IFN-*γ*) [45]. Elevated ratios of NE/SLPI in CF-UAW compared to LAW have been reported previously by Hentschel et al. assuming a greater benefit of NE inhibitors in the sinonasal than in the pulmonary compartment based on a more pronounced imbalance, than for the MMP-9/TIMP-1 ratio [16].

Furthermore, proteolytic active mediators, such as human cysteine cathepsins, are involved in lung injury and tissue remodelling in CF patients' pulmonary inflammation. So far, increased levels of cathepsins were found in sputum of CF patients, allowing their use as inflammation markers [9]. The cathepsins, including cathepsin S (CTSS), are produced by macrophages and are involved in matrix remodeling and antigen processing [10]. The acid pH-value of the airway surface liquid in CF provides an optimal condition for their activity [11]. Cathepsins cleave and inactivate antimicrobial peptides or proteins such as SLPI, which leads to an inactivation of SLPI anti-NE capacity [12].

Altogether, chronic inflammation in the CF airways is characterized by an imbalance of proteases and antiproteases, such as NE and SLPI or MMP-9 and tissue inhibitor of metalloproteinase-1 (TIMP-1). MMP-9 as a biological active enzyme is known to be released, especially in the airways, by neutrophils, macrophages, and epithelial cells in response to inflammation and takes part in the remodeling and degradation of extracellular matrix proteins [4, 13, 14]. Particularly in chronic lung disease, asthma, bronchopulmonary dysplasia, and *α*_1_-antitrypsin deficiency, this imbalance and an overproduction of MMP-9 play an important role in the pulmonary pathogenesis [13, 15]. So far, MMP-9 and TIMP-1 as its major physiological inhibitor by forming specific complexes with pro-MMP-9 were determined in the bronchopulmonary compartment of CF patients. Elevated levels of MMP-9 and TIMP-1 as well as an increase in MMP-9/TIMP-1 ratio have been reported previously in NL fluid, sputum, and bronchoalveolar lavages (BAL) [13, 16–18].

MMP-9 is known as the predominating MMP in bronchopulmonary secretions from CF patients. This may be due to the ability of NE to cleave and activate MMP-9 as well as to inactivate TIMP-1 [13, 15]. Moreover, in healthy subjects' induced sputum, higher levels of TIMP-1 were detectable when compared to CF patients, which emphasizes the relative lack of antiproteases in CF lungs [19]. As previously described from the bronchopulmonary compartment of stable CF patients, increased NE in sputum is related to increased MMP-9/TIMP-1 ratio and the implication of this an imbalance on proteolytic dysregulation has been discussed [19]. Additionally, we have recently described a correlation of TIMP-1 and MMP-9 to* P. aeruginosa* colonization of CF patients' airways [20].

At the same time, the impaired mucociliary clearance also has a considerable effect on the patients' UAW and paranasal sinuses, frequently causing chronic rhinosinusitis (CRS) and nasal polyps [21]. As a consequence, symptoms like chronic nasal congestion, rhinorrhoea with anterior and postnasal drip, mouth breathing, anosmia, facial pain, and sleep disorders affect the quality of life (QoL) [22]. Approaches including medical therapy and extensive endoscopic sinus surgery are measures to improve sinonasal disease in CF [23]. Beyond that, the defective sinonasal mucociliary clearance makes the UAW a gateway for primary pathogen colonization and a reservoir for descending infection of the lower respiratory tract [24–26]. So far, a recent series of studies found concordant strains of* P. aeruginosa *in the UAW and LAW of CF patients. Consequently, the authors postulate to treat the UAW and LAW as one airway system [21, 27, 28]. In this regard, early detection of pathogen colonization and an effective eradication by antibiotic prophylaxis or therapy may prevent subsequent descent to the LAW or exacerbations [29].

In order to preserve a good pulmonary function and to improve the QoL, particularly the treatment against a chronic infection with* P. aeruginosa* is a main focus of attention in CF care. There is evidence that a systemic intravenous- (IV-) antibiotic therapy, either applied in a more preventive elective regimen or applied symptomatically at acute pulmonary exacerbations (APE), combined with long-term nebulized antibiotic therapy benefits CF patients chronically colonized with* P. aeruginosa *[29]. The elective IV-antibiotic treatment of colonized patients for eradication and/or reduction of the pathogen burden and the resulting pulmonary inflammation belong to standards of care in many European CF centers [30, 31]. However, regimes vary regarding duration and dosage of therapy and there is no final evidence for superiority of one concept [30].

Previous studies of our group compared proteases/antiproteases relations in the UAW and LAW in a cross-sectional study [16].

The main purpose of the present longitudinal study was to analyze changes of proteases and antiproteases in sputum and NL together with changes in pathogens detected with conventional microbiological tools in both the upper and lower airway compartments. Levels of MMP-9, TIMP-1, SLPI, NE, and CTSS were quantified in NL and sputum from CF patients before and after a 14-day IV-antibiotic therapy and compared to results from healthy controls. Furthermore, we assessed the impact of the treatment on sinonasal symptoms (health-related QoL). We hypothesized that in CF patients' UAW and LAW non-invasively assessed by NL and sputum chronic imbalance of proteases and antiproteases can be adjusted after a 14-day IV-antibiotic therapy.

## 2. Patients, Materials, and Methods

### 2.1. Study Population

The prospective case control study conducted at the Jena University Hospital CF Center, Germany, included 17 CF patients who underwent 19 IV-antibiotic treatments between August 2012 and January 2013. Inclusion criteria were a diagnosis of CF confirmed by two positive sweat tests and/or a molecular genetic identification of two disease-causing* CFTR* mutations. Exclusion criteria were relevant nasal bleeding and perforation of the tympanum in general.

IV-antibiotics were administered in accordance with the current European guidelines [3] with two agents (e.g., aminoglycoside and cephalosporin or carbapenem) for 14 days. The selection of antibiotics was based on antibiotic sensitivity of pathogens cultured in sputum. NL of 20 prospectively enrolled healthy subjects served as control regarding inflammatory mediators without intervention.

Sputum samples and NL from all CF patients were collected at baseline and after approximately 14 days of treatment. Additionally, all CF patients underwent routine spirometry and biochemical blood analysis prior to therapy, according to the clinical standards in the Jena CF Center. Furthermore, patients and healthy subjects were assessed for UAW-related symptoms and health-related QoL by the Sinonasal Outcome Test 20 in its German Adapted Version (SNOT-20-GAV).

The study was approved by the Ethics Committee of the Faculty of Medicine, University of Jena, Germany (reference number: 2909/08-10). Written informed consent was obtained from each subject or their parental guardians.

### 2.2. Nasal Lavage

NL, using 10 mL of sterile isotonic saline (0.9% NaCl, Braun, Melsungen, Germany) per nostril, was performed as described previously [32]. Immediately after collection, NL fluid was either aliquoted with and without protease inhibitor (PI) (Protease Inhibitor Mix G, SERVA Electrophoresis GmbH, Heidelberg, Germany) or centrifuged for 7 min at 400 rpm. Supernatants were aliquoted with and without PI and frozen at −70°C. For cytological analysis, 5 mL of NL was added to 0.5 mL fetal calf serum (FCS, Biochrom AG, Berlin, Germany). The suspension was centrifuged for 7 min at 400 rpm. Supernatant was discarded leaving 1 mL for resuspension of the cell pellet. 100 *μ*L of FCS was added.

### 2.3. Sputum

Sputum samples were collected from patients by spontaneous expectoration. Immediately after collection, samples were diluted with four times the sputum volume of sterile phosphate buffered saline (PBS) and homogenized. Afterwards, four times the sputum volume of freshly prepared dithiothreitol (DTT) and 0.2 mL/g sputum of DNase (Roche, Basel, Switzerland) were added, vortexed for 30 seconds, and filtered. 100 *μ*L of FCS was added to 1 mL of the suspension for cytological analysis. The filtrated suspension was centrifuged for 7 min at 400 rpm. Supernatants were aliquoted with and without PI and frozen at −70°C [33].

### 2.4. Microbiology

Microbial analyses of NL and sputum collected before and after IV-antibiotic therapy were performed according to European standards. Chronic colonization was stated using the criteria published by Lee et al. [34]. The following bacteria frequently found in NL and sputum cultures were considered as part of the physiological flora of the human nasopharynx:* Neisseria* spp., alpha-hemolytic streptococci, coagulase-negative staphylococci, corynebacteria spp., stomatococci, and nonhemolytic streptococci [35, 36].

### 2.5. Cytology and Protein Concentrations

The analysis of total cell counts (TCC) and the automated cell differentiation were performed using fluorescence flow cytometry (Sysmex XE-5000, Sysmex Deutschland GmbH, Norderstedt, Germany) in Body Fluid Modus. For cytological differentiation (100 cells), cytospin preparations (100 ×g, 3 min) were prepared. Levels of total protein were measured using 3 *μ*L of NL and supernatants of sputum on an LVis Plate (SPECTROstar Omega, Omega-Data Analysis, BMG Labtech, Ortenberg, Germany) at 280 nm wavelength.

### 2.6. Inflammatory Mediators

#### 2.6.1. Multiplexed Immunoassays

Concentrations of MMP-9 and TIMP-1 (Milliplex MAP Kit, Millipore Corporation, Billerica, USA, Human MMP Panel 2 number HMMP2MAG-55K, Human TIMP Panel 1 number HTMP1MAG-54K) were measured by applying multiplexed immunoassays according to the manufacturers' instructions. In brief, all different antibody-coated beads were incubated with 25 *μ*L of NL or sputum. Sputum was diluted using assay buffer (MMP-9 1 : 20, TIMP-1 1 : 4). For detection, antibodies and streptavidin were added. Samples were measured using Bio-Plex 200 System. Results were calculated by using Bio-Plex Manager 6.0.

#### 2.6.2. ELISA

Analysis of NE, SLPI, and CTSS in NL and sputum was done in duplicate using ELISA according to the manufacturers' instructions (PMN Elastase ELISA, Milenia Biotec, Gießen, Germany, number MKEL1; SLPI ELISA, number E91312Hu; CTSS ELISA, number E91933Hu, Uscn Life science Inc., Wuhan, China). Additionally, sputum was diluted 1 : 10 for NE and CTSS detection and 1 : 100 for SLPI with assay buffer. For washing an automated washer (SLT Typ Columbus, Labtechnologies, Austria) and for detection a spectrometer FluoStar Galaxy (BMG Labtechnologies, Offenburg, Germany) were used.

### 2.7. SNOT-20-GAV

The SNOT-20-GAV is a disease-specific 20-item survey on rhinological and general complaints as well as on QoL for patients with rhinosinusitis [37, 38]. Scores were assessed before and after IV treatment and range between 0 and 5 for each item, with higher scores indicating a greater health-related burden by rhinosinusitis. In this study, the SNOT overall score with all 20 items was included for evaluation.

### 2.8. Statistical Analysis

Data was evaluated using MS Excel, IBM SPSS 21.0, and Graph Prism 6. Longitudinal values of measured parameters were compared using Wilcoxon test. Data analysis was performed using descriptive statistics, including absolute and relative frequencies, mean and standard deviation, and median and range. Correlations between measured inflammatory markers in transverse sections and clinical or serological parameters were done using Spearman's Rho. Analyzed groups were compared performing Mann-Whitney* U* test. Statistical value of *P* ≤ 0.05 was considered significant.

## 3. Results

### 3.1. Demographic Data

17 CF patients (10 female/7 male, mean age 25.1 yrs, range 8–35) who attended in the Jena University Hospital CF Center, Germany, were included. Patients received either an elective routinely IV-antibiotic treatment (18/19) or an IV treatment for acute pulmonary exacerbation (APE) (1/19). Median duration between the first and second dates within the study resulted in 15 days (range 12–23 days). The 20 healthy controls (15 female/5 male) were aged 28.5 years by mean (range: 23–48 years).

**Figure 1:**
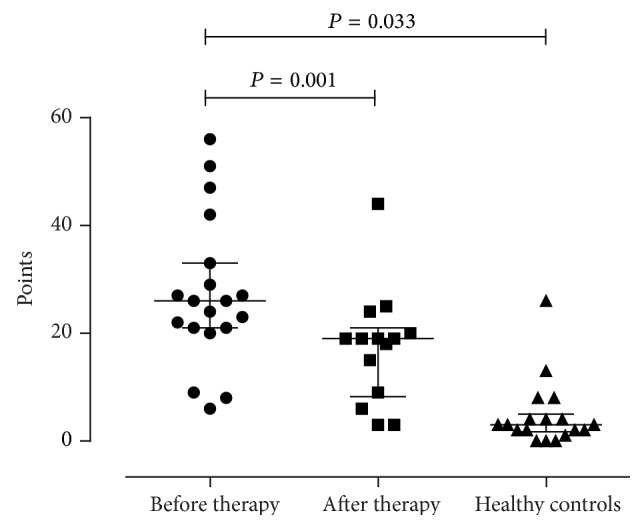
Comparison of SNOT-20-GAV scores before and after IV-antibiotic therapy in CF and in healthy controls. In CF a significant decrease was shown during therapy from a median of 26 points to 19 points (*P* = 0.001) which was still elevated (n.s.) compared to healthy controls (median: 3 points).

5 of 17 patients fulfilled the criteria for chronic rhinosinusitis (CRS) according to EPOS 2012 criteria [46]. Sinonasal symptoms SNOT-20-GAV scores decreased significantly (*P* = 0.001) during therapy from a mean of 27.3 points (median = 26; range: 6–56 points) to 17.4 points (median = 19; range: 3–44 points) as seen in Figure 1; in contrast to CF patients prior to therapy the included healthy subjects stated a mean of 4.7 points (median = 3; range = 0–26; *P* = 0.033, *r* = 0.489).

Serological inflammation markers, for example, CRP and ESR, were determined only prior to IV therapy. No significant correlations between inflammatory mediators in sputum and NL and systemic inflammation markers were found. Further clinical and serological data of included patients are presented in Tables 1 and 2.

### 3.2. Microbiological Data

At inclusion date pathogenic bacteria and/or fungi were detected in 12 (63.2%) and 16 (84.2%) out of 19 patients for NL and sputum and at exclusion date in 10 (52.6%) and 11 patients (57.9%).* P. aeruginosa* was the most commonly cultured bacterium detected in both the upper and lower airways before therapy. 42.1% of NL and 52.6% of sputum samples revealed the pathogen prior to therapy and detection rates declined to 36.8% for both sputum and NL after therapy. Whereas* S. aureus* including* MRSA* was less frequent in sputum samples (21.1% of NL and 26.3% in sputum) before therapy, none were detectable after therapy.* E. coli* was detected in 4 patients. Culture-based microbiological findings of both patients and controls before and after therapy are displayed in Table 3.

Chronic colonization of the UAW or LAW with* P. aeruginosa* was found in 4 (23.5%) and 5 (29.4%), respectively, out of 17 patients; those who were intermittently infected were 4 (23.5%) and 3 (17.6%), respectively, patients [34]. Further data are shown in Table 1. In four patients chronic colonization status could not be determined for lack of data.

### 3.3. Cytological Data and Protein Concentrations

**Figure 2:**
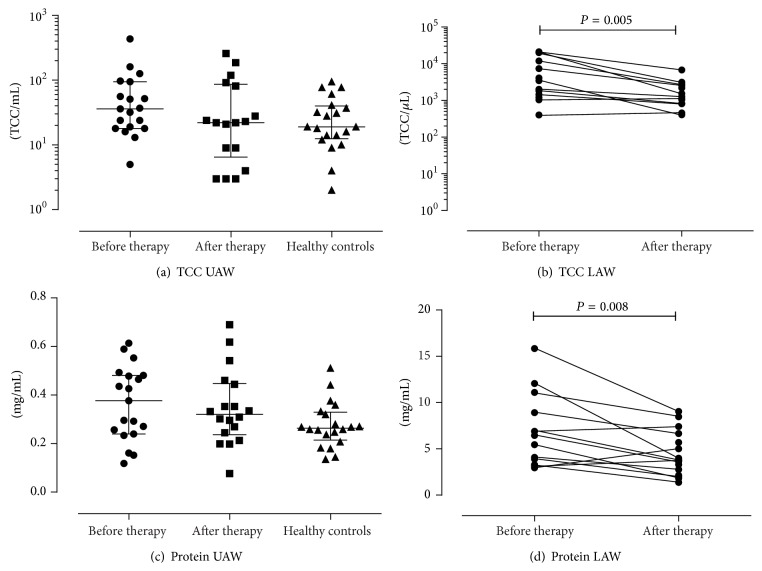
Changes of TCC and protein concentration. Both decreased in UAW and LAW after IV-antibiotic therapy, but only the decline in the LAW was shown to be statistically significant for TCC (b) and protein (d). Similar results were seen for CF patients' UAW and healthy controls (a + c).

TCC was assessed for all patients before and after therapy and decreased in both NL and sputum during therapy. However, TCC in UAW did not differ significantly between CF patients and healthy controls (Figure 2(a)). Again, the decrease of the median TCC after IV-antibiotic therapy was statistically significant only in sputum (*P* = 0.005; see Figure 2(b)). Significant positive correlations were found between TCC and MMP-9 (*r* = 0.805^UAW1^, *P* < 0.001; *r* = 0.620^UAW2^, *P* = 0.008) before and after IV therapy. Changes of TCC correlated significantly with changes of protein (*r* = 0.706^LAW1^, *P* = 0.013; *r* = 0.846^LAW2^, *P* = 0.001) at both time points. Interestingly, only after IV therapy TCC correlated significantly with MMP-9 (*r* = 0.620^UAW2^, *P* = 0.008) and protein (*r* = 0.586^UAW2^, *P* = 0.017; *r* = 0.846^LAW2^, *P* = 0.001) in both airways.

A decline of the median protein concentrations during IV-antibiotic treatment was seen for both airways (Figures 2(c) and 2(d)). However, statistical significance (*P* = 0.008) was reached only for the LAW. In healthy controls protein concentrations in NL resulted to be similar to CF patients. Changes of protein concentrations and cytology in the UAW and LAW as well as the results of healthy controls are summarized in Table 4.

### 3.4. Standardization by TCC and Protein

For standardization of the immunological markers, we divided the measured values by concentrations of TCC and protein. Calculated values did not differ significantly when related to protein concentrations. Therefore, we can exclude protein concentrations as bias. In contrast, normalization by TCC resulted in differences for all assessed parameters. Thus, cell count in secretions critically influences concentrations of inflammation markers in NL and sputum.

### 3.5. Analysis of Inflammation Markers in CF Patients before and after IV Antibiotic Therapy, Compared to Healthy Controls

**Figure 3:**
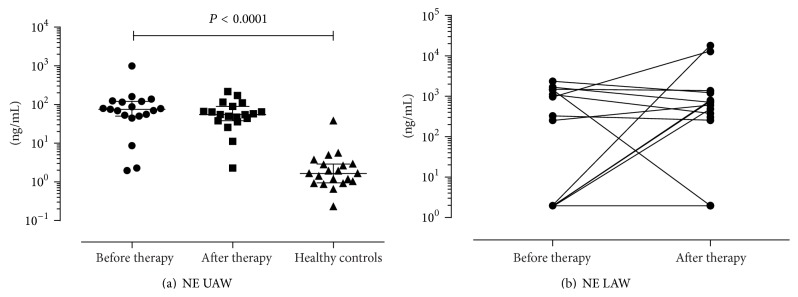
Changes of NE. Levels decreased in the UAW (a) after IV-antibiotic therapy. NE was statistically significantly lower in healthy controls compared to CF patients (*P* < 0.0001). In the LAW median levels increased after therapy, but five of seven matched pairs diminished after therapy (b).

**Figure 4:**
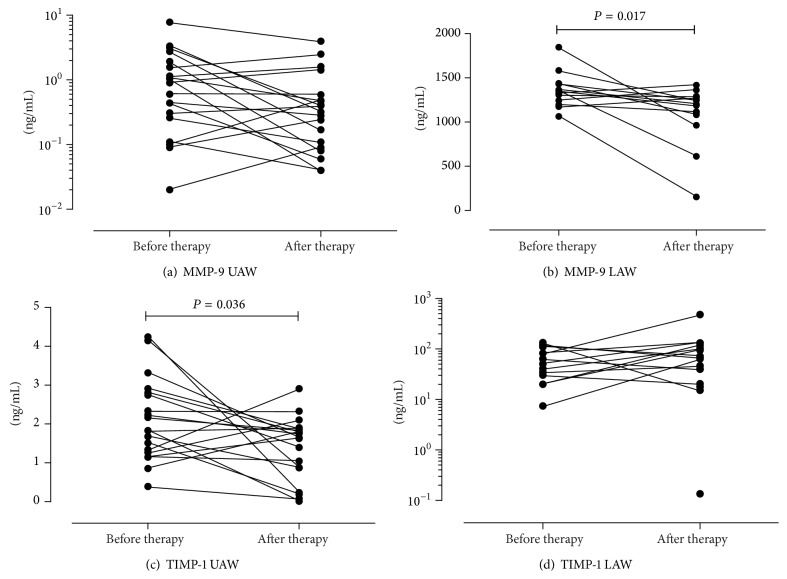
Changes of MMP-9 and its inhibitor TIMP-1. Levels of MMP-9 decreased in UAW (a) and LAW (b) after therapy; only changes in LAW reached statistical significance. Concentrations of TIMP-1 decreased significantly in the UAW (c) while levels increased in the LAW (d).

**Figure 5:**
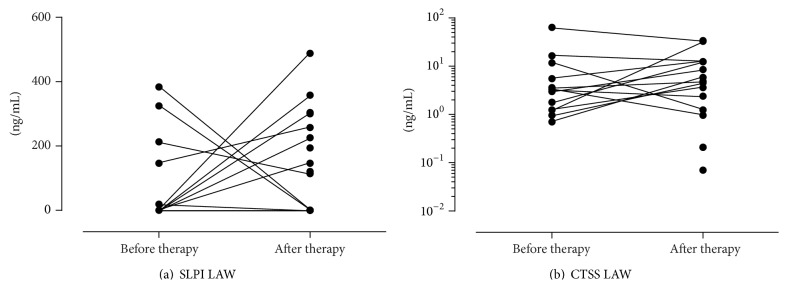
Changes of SLPI and CTSS.

**Figure 6:**
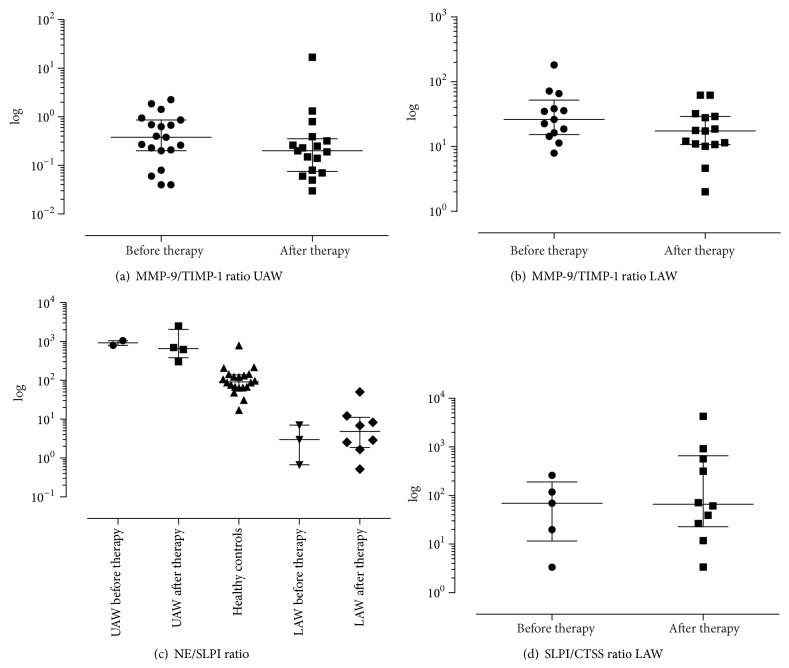
Ratios of proteases and antiproteases: MMP-9/TIMP-1 ratio (a) was higher in the LAW (b), whereas NE/SLPI ratio (c) was higher in the UAW and in healthy controls. SLPI/CTSS (d) was only calculable in the bronchial compartment for low detection rates of both parameters in the UAW. For all of these ratios in UAW and LAW changes did not reach statistical significance. 1 = before therapy; 2 = after therapy.

Regularly detected inflammation markers in UAW were NE (Figure 3(a)), TIMP-1 (Figure 4(c)), and MMP-9 (Figure 4(a)). In contrast CTSS (Figure 5(b)), TIMP-1 (Figure 4(d)), and MMP-9 (Figure 4(b)) were found consistently in LAW, whereas NE (Figure 3(b)) was only detected in 61.5% before and in 81.3% after therapy. In NL of healthy controls NE was found regularly; CTSS was detected frequently in 85% of samples. In comparison to CF samples levels of NE (see Figure 3(a)) and CTSS in NL of healthy controls were significantly lower (1.66 ng/mL and 0.04 ng/mL, resp., in comparison to 73.39 ng/mL and 0.07 ng/mL, resp., *P* < 0.001 and *P* < 0.001, resp.). SLPI was hardly detected in the UAW of CF patients as well as in healthy subjects, being more often found in LAW (see Figure 5(a)). Frequencies of detection, detection limits, median, and ranges are listed in Table 5. Only TIMP-1 decreased significantly during antibiotic therapy in UAW from 1.83 ng/mL to 1.65 ng/mL (*P* = 0.036) as shown in Figure 4(c). In LAW a significant decrease of MMP-9 (1359.7 ng/mL to 1195.9 ng/mL; *P* = 0.017) was found (Figure 4(a)). The ratio of MMP-9/TIMP-1 appeared to decline as well in NL as in sputum but did not reach statistical significance (Table 5). A significant correlation was shown between NE and the MMP-9/TIMP-1 ratio in the UAW before and after therapy (*r* = 0.681, *P* = 0.001 and *r* = 0.515, *P* = 0.035, resp.). The NE/SLPI ratio was 10-fold higher in CF patients in comparison to healthy controls (Figure 6(c)); in both compartments no significant change after IV therapy was measurable due to fewer counts of ratios. Only a calculation of the SLPI/CTSS ratio for sputum samples was done as detection frequencies and values were too low in NL. Before and after treatment MMP-9 in NL correlated significantly with NE (*r* = 0.587^UAW1^, *P* = 0.008 and *r* = 0.501^UAW2^, *P* = 0.029). Only at inclusion a significant correlation between MMP-9 and TIMP-1 (*r* = 0.605^UAW1^, *P* = 0.006) was detected.

## 4. Discussion

The airway system of CF patients is commonly infected with pathogens that cannot be effectively cleared due to the underlying ion channel defect and the resulting viscous secretions. The pathogens' virulence factors and the resulting inflammatory host response relevantly contribute to pulmonary destruction. The UAW are coming into the clinical and scientific focus as they were identified as a reservoir for initial and persistent airway colonization with pathogens like* S. aureus *and* P. aeruginosa, *that can be followed by LAW colonization, inflammation, and deterioration [25, 28]. Our previous studies assessed the correlation of colonization and inflammation in different airway compartments [16, 20, 47, 48]. Here, we assessed changes in pathogen colonization, proteases, antiproteases, and cells as well as symptoms after elective IV-antibiotic treatments primarily directed against* P. aeruginosa*.

In general, concentrations of detected proteases, antiproteases, and cells were lower in the UAW compared to LAW and even lower in the UAW of healthy controls. Fluid dilution, consistence, and origin of samples as well as processing of the materials may play a role in these differences. Otherwise, recent studies revealed different defense mechanisms in the upper and lower airway compartments; Kasper Aanaes showed that IgA plays a pronounced role in the UAW whereas a neutrophil-dominated host response with a strong oxidative burst is characteristic of the LAW first line host defense mechanisms [49, 50].

Reduction of TCC was found in NL and sputum, but only in sputum decreases reached statistical significance (*P* = 0.005) as reported earlier in our group [48]. Similar results were found in protein concentrations; decline was seen in both airway levels, but again, only in the pulmonary compartment, changes reached statistical significance (protein^LAW^: *P* = 0.008) (see Figure 2(d)). In accordance with other studies that investigated an association of LAW inflammatory mediators with lung function, we neither found a significant correlation of MMP-9 nor TIMP-1 with FEV_1_ [13, 51]. However, NE in the LAW correlated significantly with FEV_1_ (*P* = 0.033, *r* = 0.593) before treatment. As we only assessed pulmonary function prior to IV-antibiotic therapy we are not able to make a statement on how lung function correlated with proteases in NL and sputum on the long run.

Altogether, chronic lung diseases and inflammation are characterized by an imbalance of protease and antiprotease. In CF patients with bronchiectasis, elevated MMP-9 levels have been reported compared to non-CF bronchiectasis patients and healthy controls [52]. Besides elevated concentrations of MMP-9 and TIMP-1, an increased MMP-9/TIMP-1 ratios have been reported from sputum and BAL [13, 17, 18]. In our study, concentrations of MMP-9 decreased in the UAW as well as in the LAW during IV-antibiotic treatment while levels of its main inhibitor TIMP-1 attenuated in NL but even rose in sputum of CF patients. Interestingly, the reduction of MMP-9 in LAW resulted in a decline of MMP-9/TIMP-1 ratio after therapy, suggesting the ratio as a good marker for therapeutic success. The trend for reduction of MMP-9 in our study points to the inhibition of inflammation throughout the antibiotic treatment and may serve as an interesting marker to assess therapeutic effects in future studies.

Coherence between NE, MMP-9, and TIMP-1 has been described previously [13, 15, 19]. Gaggar et al. demonstrated a strong correlation between NE and MMP-9 in CF patients' sputum [15]. Due to the modified balance of MMP-9 and TIMP-1, progressive damage of lung tissue mediated by increased NE levels as well as an elevated humoral inflammation and influx of inflammatory cells is the consequence [13]. Furthermore, neutrophils can release MMP-9 in response to the proinflammatory cytokine TNF, which enhances tissue degradation. In our study MMP-9 and NE correlated significantly only in the UAW prior to and after therapy (*P* = 0.009, *r* = 0.582 and *P* = 0.031, *r* = 0.496, resp.). In contrast to Jackson et al. [19] we only found a significant correlation between NE and the MMP-9/TIMP-1 ratio in the UAW, detectable for both times of assessment (*P* = 0.001, *r* = 0.681 and *P* = 0.035, *r* = 0.515, resp.). In this respect, the previously described proteolytic imbalance in the LAW also has to be regarded for the UAW, which underlines the need to look upon the CF patients' upper and lower airways as one airway system.

Our findings confirmed the chronic neutrophil-dominated pulmonary inflammation in CF resulting in higher levels of NE in the airway surface liquid not only being relevant in LAW, but also affecting the UAW [16]. In our patients, NE in NL prior to therapy was 44-fold increased when compared to healthy subjects (*P* < 0.001). This accords well with LAW data from Gaggar et al. who reported a 40-fold increased activity of NE in sputum of CF patients compared to healthy controls [15]. Within the IV-antibiotic treatment, levels of NE decreased in NL, different from our results obtained in a previous study [47]. This difference possibly is caused by a shorter observation period of 6 days in the preceding report, compared to 14 days in the present study. Unlike other publications demonstrating a significant decrease of NE in sputum after antibiotic therapy [40], median NE levels redoubled in our study. However, regarding matched values for NE before and after therapy, in five of seven patients the enzyme decreased during treatment and the huge increase in the remaining two patients cause this surprising increase of medians (see Figure 3(b)). Explanation may be that NE can be bound within neutrophil-extracellular traps (NETs), which are part of the innate immunity composed of granule and nuclear constituents, for example, DNA. NETs are regularly found in sputum of CF patients and are released by activated neutrophils [53]. Due to the routine usage of DNAse in CF patients NETs can be cleared leading to elevated levels of NE [54]. As we used DNAse in processing of sputum, more NE may be liberated and increased concentrations can be measured.

Previously Weldon et al. reported that SLPI is susceptible to proteolytic degradation by NE in chronic infection whereby it neutralizes the anti-NE capacity of SLPI [55]. The imbalance may be enhanced by high burdens of NE in ASL which can overwhelm and inactivate SLPI [8]. Additionally, SLPI as an immunomodulatory protein is capable of decreasing MMP-9 in monocytes [56]. Low detection frequencies of SLPI in all assessed materials are a limitation of the present study. As induced sputum was not taken from controls, a comparison to SLPI levels in the healthy could not be performed. However, during therapy, levels of SLPI increased in sputum whereas its concentrations in NL of CF patients as those of healthy controls remained low, if detectable.

Also CTSS has the potential to cleave and inactivate SLPI which further increases NE levels and facilitates bacterial colonization and infection [9, 57]. Lecaille et al. assessed the cleavage of surfactant protein A, which belongs to the innate immunity, system by CTSS which also facilitates infections by pathogens like* P. aeruginosa* [58]. However, in healthy lungs, cathepsins have not been detected routinely, but they may be stimulated by different mediators, such as IFN-*γ* or IL-13 [9]. Whereas our findings of elevated cathepsin levels in sputum confirm earlier reports, detection frequencies and levels of CTSS in NL compared to healthy controls were rather low.

Interestingly, inverse results of calculated protease/antiprotease ratios were found in both airway levels. While MMP-9/TIMP-1 ratios were higher in the LAW than in the UAW before and after therapy (65-fold: 26.1/0.4 and 87-fold: 17.4/0.2, resp.), NE/SLPI ratios were higher in the sinonasal compartment compared to the lung (306-fold: 919.1/3.0 and 134-fold: 658.1/4.9, resp.) (Figure 1). These MMP-9/TIMP-1 and NE/SLPI ratios accord well with recent findings from Hentschel et al. who additionally detected elevated SLPI/CTSS values in LAW compared to UAW (16-fold) [16]. As expected, MMP-9/TIMP-1 ratios showed a trend to decrease during systemic treatment in both airway levels (UAW: 1.9-fold, LAW: 1.5-fold, not statistically significant). In this regard, it is remarkable that even clinical stable CF patients with mild pulmonary disease revealed an imbalance of the MMP-9/TIMP-1 ratio in BAL indicating the contribution of the proteases in the chronic inflammatory process in CF lung disease [17]. While in our patients the NE/SLPI ratio in the UAW was 1.4-fold higher before therapy, inverse results were shown for the LAW where the ration was higher after therapy (1.6-fold). Compared to healthy results, NE/SLPI ratio was 10-fold elevated in the sinonasal compartment.

This paper for the first time compares changes in UAW and LAW colonization pattern after IV-antibiotic treatment. Before and after therapy* P. aeruginosa *was frequently detected in the UAW and LAW of the patients with history of chronic colonization with this pathogen (see Table 3) indicating that the IV-antibiotic treatment may reduce but not eradicate colonization and reduce the resulting inflammatory response. Further analyses assessing the upper and lower airways' microbiome by molecular methods with a comparable study design are of high scientific interest.

Only few studies examined changes of sinonasal symptoms and health-related QoL during targeted therapy in CF patients. In this regard, Mainz et al. reported a significant reduction of symptoms assessed by the SNOT-20-GAV scores after sinonasal inhalation with dornase alfa or tobramycin [59, 60]. Savastano et al. evaluated the postoperative outcome of CF patients undergoing sinonasal surgery for CRS and nasal polyposis using the SNOT-22 score and also concluded a positive impact on QoL [23]. In our study, a remarkable improvement of SNOT-20-GAV scores was found after IV-antibiotic treatment. However, scores still were significantly higher when compared to those of healthy controls (see Figure 1). In conclusion, elective IV-antibiotic treatment does not only improve LAW inflammation; it also reduces subjective symptoms of the UAW and general QoL.

## 5. Conclusion

The present paper for the first time demonstrates changes in UAW and LAW proteases and antiproteases (NE, SLPI, CTSS, MMP-9, and TIMP-1) and pathogen colonization after IV-antibiotic therapy. Further analyses on changes of the protease/antiprotease imbalance especially in the paranasal sinuses and its correlation to changes in the microbiome are of special interest.

## Figures and Tables

**Table 1:** Clinical, microbiological, and serological characteristics of included patients and healthy controls.

Nominal variables	*N*	Absolute frequency
Cystic fibrosis patients		
Gender (female)	17	10 (58.8%)
Nasal polyps	17	5 (29.4%)
History of sinonasal surgery	17	6 (35.3%)
Chronic rhinosinusitis	17	5 (29.4%)
Allergy		
*Aspergillus fumigatus *	17	7 (41.2%)
House dust mite		3 (17.6%)
Grass pollen		2 (11.8%)
Pet hair (cat/dog)		2 (11.8%)/1 (5.9%)
ABPA	17	2 (11.8%)
Allergic rhinitis	17	2 (11.8%)
Diabetes mellitus	17	5 (29.4%)
IV-antibiotics	19	
Tobramycin		18 (94.7%)
Ceftazidime		9 (47.4%)
Tazobactam/piperacillin		4 (21.1%)
Colistin		1 (5.3%)
Meropenem		6 (31.6%)
Therapy		
Current azithromycin	17	12 (70.6%)
Current oral antibiotics		9 (52.9%)
Current inhalative antibiotics		16 (94.1%)
Recombinant DNAse		10 (58.8%)
Nasal topical bronchial steroids		8 (47.1%)
Current oral antimycotics		10 (58.8%)
Chronic colonization of UAW with^∗1^		
*P. aeruginosa *permanent	17	4 (23.5%)
*P. aeruginosa* intermittent		4 (23.5%)
Chronic colonization of LAW with^∗1^		
*P. aeruginosa *permanent	17	5 (29.4%)
*P. aeruginosa *intermittent		3 (17.6%)
UAW: detection of ^∗2^		12 (63.2%)
*P. aeruginosa * (mucoid)	19	6 (31.6%)
*P. aeruginosa * (nonmucoid)		4 (21.1%)
*S. aureus *		2 (10.5%)
*MRSA *		2 (10.5%)
LAW: detection of ^∗2^		16 (84.2%)
*P. aeruginosa * (mucoid)	19	9 (47.4)
*P. aeruginosa * (nonmucoid)		7 (36.8%)
*S. aureus *		3 (15.8%)
*MRSA *		2 (10.5%)
*P. aeruginosa *serum antibodies positive:		8 (50.0%)
Alkaline protease/borderline	16	8 (50%)/2 (12.5%)
Exotoxin A/borderline		9 (56.3%)/1 (6.3%)
Elastase/borderline		9 (56.3%)/1 (6.3%)

Healthy controls		
Gender (female)	20	15 (75%)
Allergic rhinitis		1 (5%)
Allergy in general		4 (20%)
Postnasal drip		4 (20%)
History of ORL surgery		5 (25%)
Snore		3 (15%)

^∗1^Permanent and intermittent colonization were stated using the criteria published by Lee et al. [34]. Chronic colonization is defined if 50% or more of cultures within the last year were found positive and intermittent if less than 50% of cultures within the last year were found positive.

^∗2^At inclusion date.

**Table 2:** Clinical and serological characteristics of included patients and healthy controls.

Metric and ordinal variables	*N*	Mean ± SD	Median	Range
Age (yrs)	17	25.5 ± 7.1	25.0	8–35
BMI (kg/m^2^)	17	19.5 ± 3.6	19.2	14.7–29.3
FEV1 (l)/(% predicted)	17	1.9 ± 1.3 (57.9 ± 38.1)	1.3 (38.1)	0.8–5.7 (25–141)
MEF75/25 (l)/(% predicted)	13	1.4 ± 1.4 (36.9 ± 40.4)	0.7 (19.0)	0.3–4.6 (8.0–139.8)
ESR (mm/h)	18	34.3 ± 26.0	24.0	2–85
CRP (mg/l)	19	18.6 ± 30.5	5.7	0.5–108.1
Total IgG (g/l)	19	16.5 ± 4.7	17.4	9.5–27.8
Total IgE (kU/l)	18	324.3 ± 529.0	62.7	6.4–1568
SNOT-GAV-20				
Prior to therapy	19	27.3 ± 13.7	26.0	6–56
After therapy	19	17.4 ± 10.6	19.0	3–44

Healthy controls				
Age (yrs)	20	28.2 ± 7.4	25.0	23–48
BMI (kg/m^2^)		21.9 ± 3.1	21.0	17.7–28.3
SNOT-20-GAV		4.7 ± 11.8	3.0	0–26

**Table 3:** Culture-based detection of pathogens in UAW and LAW before and after IV-AB therapy.

	Age/gender	Site	Before therapy	After therapy
Pat. 1	35 yrs/m No chronic colonization evaluable due to lack of data	UAW	*P. aeruginosa* (mucoid) *S. aureus *	*P. aeruginosa* (mucoid) *P. aeruginosa *
		LAW	*P. aeruginosa* (mucoid) *S. aureus* *Enterococcus aureus *	*P. aeruginosa* (mucoid) Yeast

Pat. 2	32 yrs/f	UAW	*MRSA *	n.m.
		LAW	*MRSA* *Aspergillus flavus *	Culture negative

Pat. 3	30 yrs/f Permanent P.a.^+^ UAW/LAW Intermittent S.a.^+^ UAW/LAW (MRSA)	UAW	*P. aeruginosa* (mucoid) *MRSA* *Klebsiella oxytoca *	*P. aeruginosa* (mucoid) *P. aeruginosa *
		LAW	*P. aeruginosa* (mucoid) *P. aeruginosa* *MRSA* *Enterococcus *spp.	*P. aeruginosa* (mucoid) *P. aeruginosa* Yeast

Pat. 4	30 yrs/m^*^	UAW	*E. coli *	*P. aeruginosa* (mucoid) *P. aeruginosa *
		LAW	*P. aeruginosa* (mucoid) *P. aeruginosa* *S. aureus* *E. coli *	*P. aeruginosa* (mucoid) *P. aeruginosa* Yeast

Pat. 5	27 yrs/m Permanent P.a.^+^ UAW/LAW Permanent S.a.^+^ LAW Intermittent S.a.^+^ UAW	UAW	*P. aeruginosa* (mucoid) *S. aureus* *Serratiamarcescens *	*Comamonas testosteroni*/*P. alcaligenes *
		LAW	*P. aeruginosa* (mucoid) *S. aureus *	*P. aeruginosa* (mucoid) Yeast

Pat. 6	25 yrs/f Intermittent P.a.^+^ LAW	UAW	Culture negative	Culture negative
		LAW	*P. aeruginosa* (mucoid) *P. aeruginosa* *E. coli *	*P. aeruginosa* *E. coli *

Pat. 7	23 yrs/f Intermittent P.a.^+^ UAW/LAW	UAW	Culture negative	*P. aeruginosa *
		LAW	Culture negative	Culture negative

Pat. 8	23 yrs/f Permanent S.a.^+^ UAW/LAW	UAW	Culture negative	Culture negative
		LAW	Yeast	Yeast

Pat. 9	18 yrs/m^*^	UAW	n.m.	Culture negative
		LAW	*S. aureus *	Culture negative

Pat. 10	35 yrs/f Permanent P.a.^+^ LAW Intermittent P.a.^+^ UAW	UAW	*P. aeruginosa *	n.m.
		LAW	*P. aeruginosa* (mucoid) *P. aeruginosa* Yeast	n.m.

Pat. 11	31 yrs/f Intermittent S.a.^*^ LAW?	UAW	*E. coli* *Proteus mirabilis *	*E. coli *
		LAW	*E. coli* *Proteus mirabilis* *Aspergillus fumigatus* Yeast	*E. coli* *Aspergillus fumigatus* *P. fluorescens *

Pat. 12	15 yrs/f Intermittent P.a.^+^ UAW/LAW Permanent S.a.^+^ UAW/LAW	UAW LAW	*P. aeruginosa* *P. aeruginosa *	Culture negative Culture negative

Pat. 13	25 yrs/f Permanent P.a.^+^ UAW/LAW	UAW	*P. aeruginosa* (mucoid) *Enterococcus faecalis *	Culture negative
		LAW	*P. aeruginosa* (mucoid) *P. aeruginosa* *Aspergillus fumigatus *	Culture negative

Pat. 14	30 yrs/m Permanent P.a.^+^ UAW/LAW	UAW	*E. coli* *P. fluorescens *	n.m.
		LAW	*E. coli* *P. fluorescens* *S. viridans * Yeast	*Aspergillus fumigatus* *Klebsiella oxytoca* *P. putida* *Enterobacter cloacae* *Yeast *

Pat. 15	8 yrs/m Intermittent P.a.^+^ UAW Permanent S.a.^+^ UAW/LAW	UAW LAW	Culture negative *Haemophilus parainfluenzae *	Culture negative Culture negative

Pat. 16	22 yrs/f^*^			
	Course 1	UAW	*P. aeruginosa* (mucoid) *P. aeruginosa *	*P. aeruginosa* (mucoid) *P. aeruginosa *
		LAW	*P. aeruginosa* (mucoid) *P. aeruginosa* *Aspergillus fumigatus *	*P. aeruginosa* (mucoid) *P. aeruginosa *
	Course 2	UAW	n.m.	*P. aeruginosa* (mucoid) *P. aeruginosa *
		LAW	n.m.	*P. aeruginosa* *Streptococcus pneumoniae *

Pat. 17	24 yrs/m^*^			
	Course 1	UAW	*P. aeruginosa* (mucoid) *P. aeruginosa *	*P. aeruginosa* (mucoid) *P. aeruginosa* Enterococci
		LAW	*P. aeruginosa* (mucoid) Yeast	Yeast
	Course 2	UAW	n.m.	n.m.
		LAW	n.m.	n.m.

f = female, m = male, and n.m. = not measured.

^*^Chronic colonization status not evaluable due to lack of data.

**Table 4:** Changes of protein concentrations and cytology before and after IV-AB treatment and comparison to findings in healthy controls.

Analyte (unit)	Concentrations						
	Median			Range			*P *
	Controls	CF prior to therapy	CF after therapy	Controls	CF prior to therapy	CF after therapy	
NL (UAW)							
Total protein (mg/mL)	0.26	0.38	0.32	0.14–0.51	0.12–0.61	0.08–0.69	0.734^*^ 0.073°
TCC (TCC/*μ*L)	19	36	22	2–95	5–433	3–259	0.178^*^ 0.078°
PMN (%)	78.5	69	88	42–100	0–90	45–100	0.011^*^ 0.044°
MN (%)	21	31	12	0–58	10–100	0–55	0.012^*^ 0.038°
Sputum (LAW)							
Total protein (mg/mL)		6.5	3.8		3.0–15.9	1.4–9.1	0.008^*^
TCC (TCC/*μ*L)		3452	1272		400–21234	408–6788	0.005^*^
PMN (%)		84	88		26–95	39–96	0.636^*^
MN (%)		16	12		6–74	4–61	0.636^*^

^*^ *P* value between CF prior to and after therapy; °*P* value between CF prior to therapy and healthy controls in UAW.

**Table 5:** Inflammation markers and ratios in controls and CF patients before and after IV-AB treatment.

					Inflammatory marker or ratio concentrations						
Analyte	DL	Detection frequency (%)			Median			Range			*P *
		Controls	CF prior to therapy	CF after therapy	Controls	CF prior to therapy	CF after therapy	Controls	CF prior to therapy	CF after therapy	
NL (UAW)											
NE (ng/mL)	1.98	100.0	89.5	100.0	1.7	73.4	55.6	0.2–38.0	2.3–990.0	2.3–233.6	0.374^*^ 0.0000°
SLPI (ng/mL)	0.01	30.0	10.5	21.1	0.01	0.01	0.01	0.01–0.06	0.01–0.17	0.01–0.15	1.000^*^ 0.235°
CTSS (ng/mL)	0.07	85.0	5.3	21.1	0.04	0.07	0.07	0.00–0.24	0.07–0.14	0.07–1.08	0.125^*^ 0.0004°
MMP-9 (ng/mL)	0.04		100.0	100.0		0.9	0.32		0.02–7.8	0.04–3.96	0.106^*^
TIMP-1 (ng/mL)	0.01		100.0	100.0		1.8	1.7		0.4–4.2	0.01–2.9	0.036^*^
MMP-9/TIMP1		n.m.	100.0	89.5	n.m.	0.38	0.20	n.m.	0.0–2.3	0.03–17.0	0.359^*^
NE/SLPI		100.0	10.5	21.1	91.7	919.1	658.1	16.9–780.1	791.0–1047.1	301.8–2510.8	n.mb.
SLPI/CTSS			—	—	—	—	—	—	—	—	—
Sputum (LAW)											
NE (ng/mL)	1.98		61.5	81.3		328.8	697.4		2.3–2366.5	2.3–17987.2	0.791^*^
SLPI (ng/mL)	0.01		38.5	62.5		0.01	133.4		0.01–383.6	0.01–487.9	0.432^*^
CTSS (ng/mL)	0.07		100.0	93.8		3.3	4.7		0.7–63.8	0.1–33.9	0.376
MMP-9 (ng/mL)	0.04		100.0	100.0		1359.7	1195.9		1063.2–1848.0	153.5–1417.5	0.017^*^
TIMP-1 (ng/mL)			100.0	100.0		50.7	66.0		7.5–134.2	0.14–480.8	0.191^*^
MMP-9/TIMP1			100.0	93.8		26.1	17.4		7.9–181.8	2.0–62.2	0.094^*^
NE/SLPI			23.1	50.0		3.0	4.9		0.7–7.1	0.5–50.2	n.mb.
SLPI/CTSS			38.5	62.5		60.6	37.3		3.4–117.9	3.4–71.3	1.000^*^

^*^ *P* value between CF prior to and after therapy, °*P* value between CF prior to therapy and healthy controls in UAW, DL = detection limit, n.m. = not measured, and n.mb. = not measureable.

## Abbreviations

AB: Antibiotics
ABPA: Allergic bronchopulmonary aspergillosis
APE: Acute pulmonary exacerbation
ASL: Airway surface liquid
BMI: Body mass index
CF: Cystic fibrosis
CFTR: Cystic fibrosis transmembrane conductance regulator
COPD: Chronic obstructive lung disease
CrP: C-reactive protein
CRS: Chronic rhinosinusitis
CTSS: Cathepsin S
DL: Detection limit
DNA: Desoxyribonucleic acid
DTT: Dithiothreitol
ELISA: Enzyme linked immunosorbent assay
ENT: Ears, nose, and throat
ESR: Erythrocyte sedimentation rate
FCS: Fetal calf serum
FEV1: Forced expiratory pressure in 1 second
IFN: Interferon
Ig: Immune globulin
IL: Interleukin
IV: Intravenous
LAW: Lower airways
MEF75/25: Mean exploratory flow
MMP: Matrix metalloproteinase
MN: Mononuclear leucocytes
*MRSA*: Methicillin-resistant* Staphylococcus aureus*
NE: Polymorphonuclear neutrophil elastase
NL: Nasal lavage
*P. aeruginosa*, P.a.: * Pseudomonas aeruginosa*
PBS: Phosphate buffer saline
PCR: Polymerase chain reaction
PI: Protease inhibitor
PMN: Polymorphonuclear leukocytes
*S. aureus*: * Staphylococcus aureus*
SLPI: Secretory leukocyte protease inhibitor
SNOT-20-GAV: Sino-Nasal Outcome Test 20 German Adapted Version
TCC: Total cell count
TIMP: Tissue inhibitor of metalloproteinase
UAW: Upper airways.
